# Supplementary material for: Causal interplay between lactose intolerance and gut microbiota: a combined bidirectional Mendelian randomization and in vivo validation study
Source: Front Nutr. 2026 Jun 1;13:1803337. doi: 10.3389/fnut.2026.1803337 (PMC13265576; doi:10.3389/fnut.2026.1803337)
Supplement: Supplementary file 1 [file Data_Sheet_1.zip › supplementary materials/Reverse/Batch analysis results 20250616220454/leave one out cross validation/finngen_R12_E4_LACTONAS.ebi-a-GCST90027462.pdf]

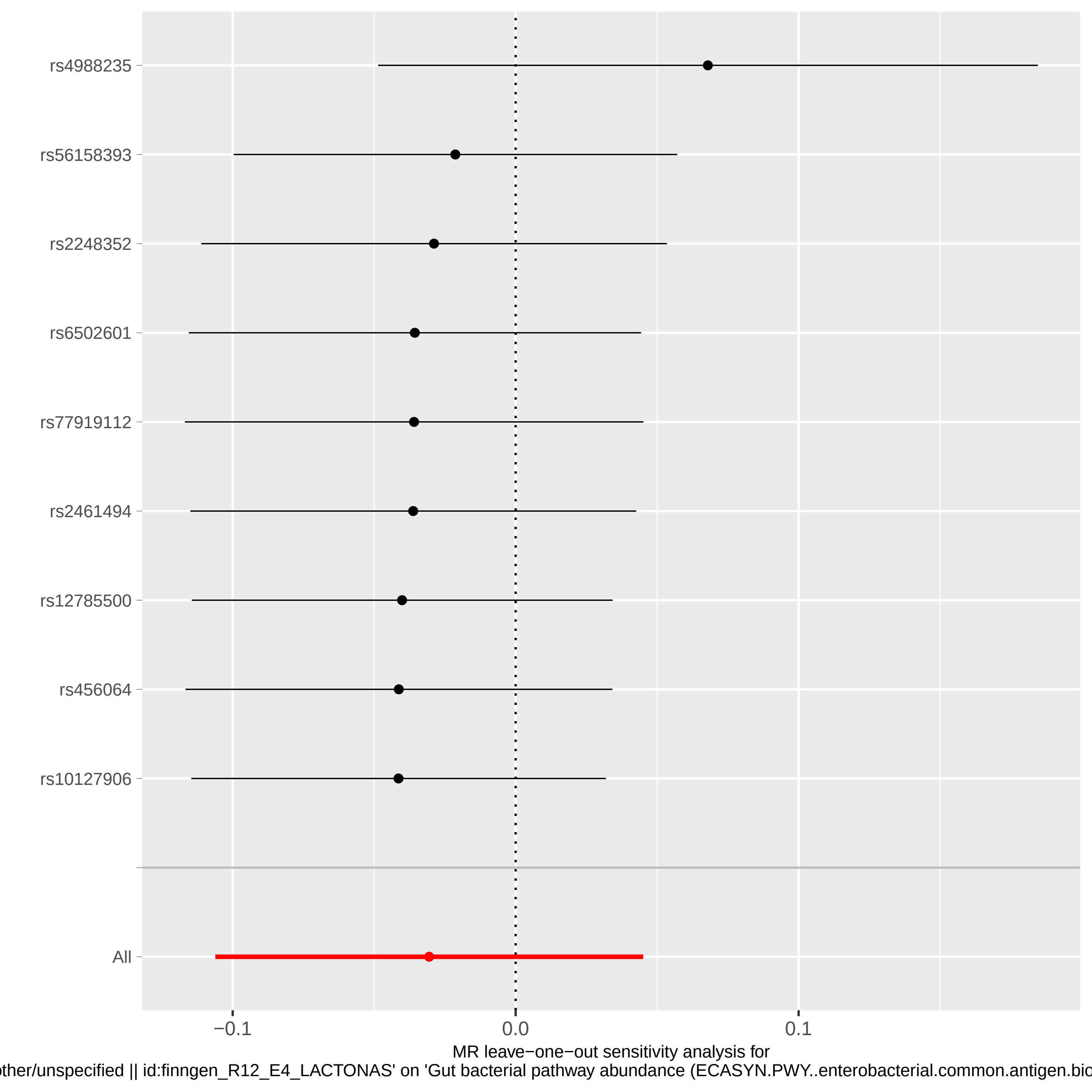

rs4988235
rs56158393
rs2248352
rs6502601
rs77919112
rs2461494
rs12785500
rs456064
rs10127906
All
−0.1
0.0
0.1
MR leave−one−out sensitivity analysis for
other/unspecified || id:finngen_R12_E4_LACTONAS' on 'Gut bacterial pathway abundance (ECASYN.PWY..enterobacterial.common.antigen.bio
